# Development and Validation of a Protein Array for Detection of Antibodies against the Tick-Borne Pathogen Borrelia miyamotoi

**DOI:** 10.1128/spectrum.02036-22

**Published:** 2022-10-31

**Authors:** Dieuwertje Hoornstra, Olga A. Stukolova, Ludmila S. Karan, Denis S. Sarksyan, Nadezhda M. Kolyasnikova, Mikhail L. Markelov, Anna S. Cherkashina, Anna S. Dolgova, Anna E. Sudina, Marina I. Sokolova, Alexander E. Platonov, Joppe W. Hovius

**Affiliations:** a Center for Experimental and Molecular Medicine, Amsterdam University Medical Centers, Academic Medical Center, Amsterdam, The Netherlands; b Central Research Institute of Epidemiology, Moscow, Russia; c Izhevsk State Medical Academy, Izhevsk, Russia; d Chumakov Federal Scientific Center for Research and Development of Immune-and-Biological Products, Russian Academy of Sciences, Moscow, Russia; e Izmerov Research Institute of Occupational Health, Moscow, Russia; f St. Petersburg Pasteur Institute of Epidemiology and Microbiology, Saint Petersburg, Russia; g Amsterdam Infection & Immunity Institute, Amsterdam University Medical Centers, Academic Medical Center, Amsterdam, The Netherlands; Tainan Hospital, Department of Health, Executive Yuan

**Keywords:** *Borrelia miyamotoi*, relapsing fever *Borrelia*, *Borrelia miyamotoi* disease, hard-tick-borne relapsing fever, serology

## Abstract

Current serological tests for the emerging tick-borne pathogen Borrelia miyamotoi lack diagnostic accuracy. To improve serodiagnosis, we investigated a protein array simultaneously screening for IgM and IgG reactivity against multiple recombinant B. miyamotoi antigens. The array included six B. miyamotoi antigens: glycerophosphodiester phosphodiesterase (GlpQ), multiple variable major proteins (Vmps), and flagellin. Sera included samples from cases of PCR-proven Borrelia miyamotoi disease (BMD), multiple potentially cross-reactive control groups (including patients with culture-proven Lyme borreliosis, confirmed Epstein-Barr virus, cytomegalovirus, or other spirochetal infections), and several healthy control groups from regions where *Ixodes* is endemic and regions where it is nonendemic. Based on receiver operating characteristic (ROC) analyses, the cutoff for reactivity per antigen was set at 5 μg/mL for IgM and IgG. The individual antigens demonstrated high sensitivity but relatively low specificity for both IgM and IgG. The best-performing single antigen (GlpQ) showed a sensitivity of 88.0% (95% confidence interval [CI], 78.9 to 93.5) and a specificity of 94.2% (95% CI, 92.7 to 95.6) for IgM/IgG. Applying the previous published diagnostic algorithm—defining seroreactivity as reactivity against GlpQ and any Vmp—revealed a significantly higher specificity of 98.5% (95% CI, 97.6 to 99.2) but a significantly lower sensitivity of 79.5% (95% CI, 69.3 to 87.0) for IgM/IgG compared to GlpQ alone. Therefore, we propose to define seroreactivity as reactivity against GlpQ and any Vmp or flagellin which resulted in a comparable sensitivity of 84.3% (95% CI, 74.7 to 90.8) and a significantly higher specificity of 97.9% (95% CI, 96.9 to 98.7) for IgM/IgG compared to GlpQ alone. In conclusion, we have developed and validated a novel serological tool to diagnose BMD that could be implemented in clinical practice and epidemiological studies.

**IMPORTANCE** This paper describes the protein array as a novel serological test for the diagnosis of Borrelia miyamotoi disease (BMD), by reporting the methodology, the development of a diagnostic algorithm, and its extensive validation. With rising numbers of ticks and tick bites, tick-borne diseases, such as BMD, urgently deserve further societal and medical attention. B. miyamotoi is prevalent in *Ixodes* ticks across the northern hemisphere. Humans are exposed to, and infected by, B. miyamotoi and develop BMD in Asia, in North America, and to a lesser extent in Europe. However, the burden of infection and disease remains largely unknown, due to the noncharacteristic clinical presentation, together with the lack of awareness and availability of diagnostic tools. With this paper, we offer a novel diagnostic tool which will assist in assessing the burden of disease and could be implemented in clinical care.

## INTRODUCTION

Borrelia miyamotoi is an emerging tick-borne pathogen of the relapsing fever *Borrelia* (RFB) group and shares its *Ixodes* vector with other tick-borne pathogens, including Borrelia burgdorferi *sensu lato*, tick-borne encephalitis virus, Anaplasma phagocytophilum, and *Ehrlichia* and *Babesia* species (1–6). The B. miyamotoi infection rate ranges from 0.7 to 2.8% in the main questing vectors Ixodes scapularis, I. pacificus, I. ricinus, and I. persulcatus (7–9). B. miyamotoi causes an acute febrile illness and flu-like symptoms in immunocompetent patients, referred to as Borrelia miyamotoi disease (BMD), and a meningoencephalitis in immunocompromised individuals (10–14). To date, hundreds of well-described disease cases have been published in Asia, North America, and Europe (9). Nevertheless, the burden of infection and disease remains unknown, due to low awareness among the general public and physicians, a noncharacteristic and possibly self-limiting clinical presentation, and, importantly, limited diagnostic tools (10).

Current diagnosis of BMD, during the febrile disease phase and prior to antibiotic treatment, is based on molecular analysis of blood or cerebrospinal fluid (CSF) samples. PCR detection of B. miyamotoi is often aimed at amplification of 16S rRNA or glycerophosphodiester phosphodiesterase (*glpQ*) or flagellin B (*flaB*) gene fragments. Due to the relatively short spirochetemia (15), PCR detection of B. miyamotoi is applicable only in the acute phase of disease or during a febrile relapse (10, 16).

After the development of a humoral immune response, BMD can be diagnosed by the detection of specific anti-B. miyamotoi IgM and/or IgG antibodies. Serological assays, including enzyme-linked immunosorbent assay (ELISA), Western blot analysis, and Luminex based assays, have been developed to determine seroreactivity against the RFB specific antigen GlpQ. This immunoreactive protein is highly conserved in the RFB group, including B. miyamotoi, but is absent in the B. burgdorferi s.l. group. Therefore, it can be used to distinguish between B. miyamotoi and B. burgdorferi sensu lato in regions where no other RFB are prevalent. Human anti-GlpQ IgM antibodies peak between 11 and 20 days after onset of disease (DOD), while IgG antibodies peak between 21 and 50 DOD (17). The sensitivity of the GlpQ-based assay ranges from 54 to 97% for IgM and 38 to 87% for IgG, and the specificity ranges from 97 to 100% for IgM and 92 to 100% for IgG (17–19).

In order to increase the diagnostic accuracy of serologic testing, the diagnostic potential of antibodies against other antigens has been explored (17, 20–22). IgM and IgG antibodies against different variable major proteins (Vmps) have been detected in acute- and late-phase BMD patient sera (22, 23), comprising variable small proteins (Vsps) and variable large proteins (Vlps; divided into Vlp-α, Vlp-γ, and Vlp-δ subfamilies). The combined diagnostic algorithm, including antibodies against GlpQ and any of the Vmps, increased the sensitivity to 79% for IgM and to 87% for IgG (17). The specificity of this algorithm—using a limited number of healthy and disease controls—was found to be 100% for IgM and 98% for IgG (17). In addition, two-tier testing by ELISA and confirmatory Western blotting against the same antigens, as is the approach in the diagnosis of Lyme borreliosis (LB), diminished the number of false-positive results (17). Although detection of specific Vmp antibodies has the potential to improve the diagnostic accuracy of BMD, several diagnostic Vmp markers might be needed for different genotypes of B. miyamotoi. Additionally, cross-reactive antibodies have been indicated in early phase LB-patients (24). A likely explanation for this is the homology of Vlps with the B. burgdorferi sensu lato outer membrane protein VlsE (Vmp-like sequence expressed) (24). Conversely, sera from BMD patients may cross-react in commercially available Lyme borreliosis diagnostic tests (25, 26). Therefore, a more efficient method with a higher diagnostic accuracy is needed. Consequently, a protein array was developed to diagnose BMD by detecting IgM and IgG antibody responses against recombinant consensus variants of known B. miyamotoi antigens.

## RESULTS

### Antibody response against Borrelia miyamotoi antigens in BMD patients.

**FIG 1:**
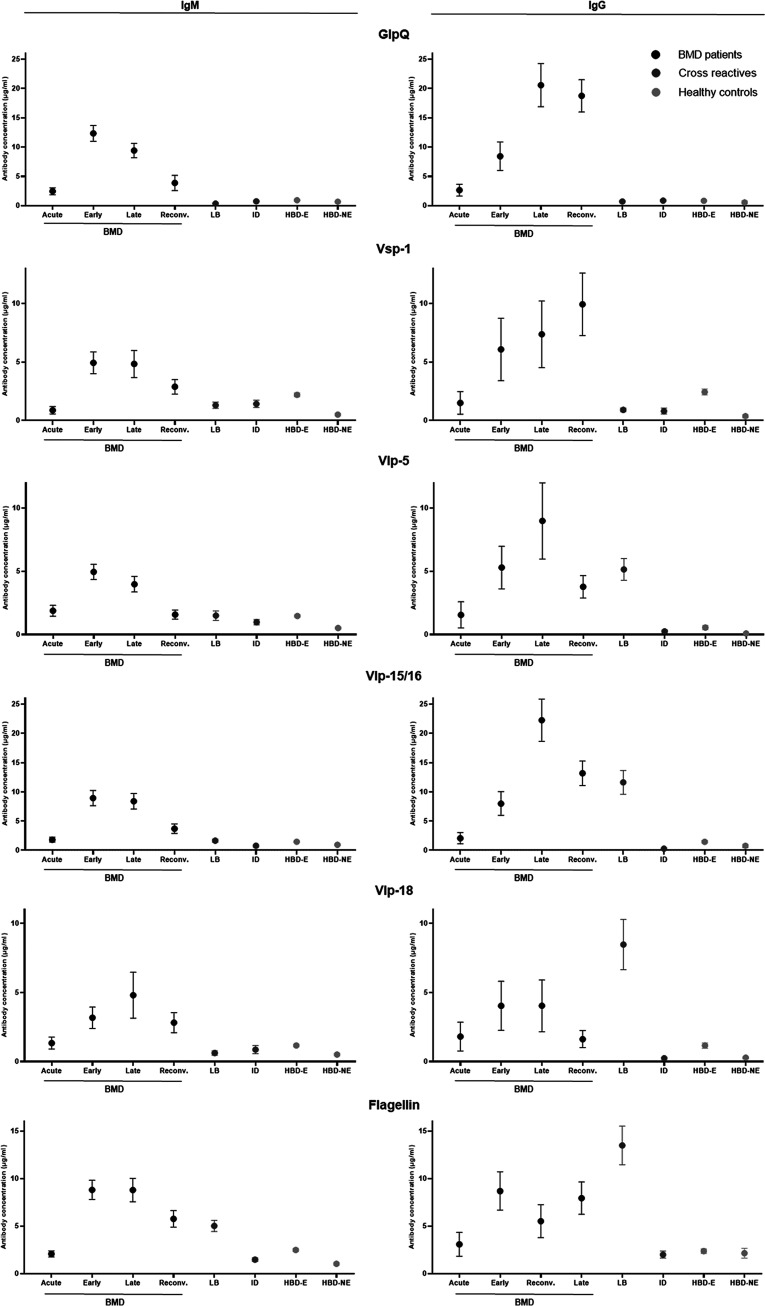
Absolute antibody response in the protein array against recombinant B. miyamotoi antigens in BMD patients and control groups. Mean values with standard errors of the means for IgM and IgG concentrations in acute-phase (0 to 4 days after disease onset), early-convalescent-phase (5 to 25 days after disease onset), late-convalescent-phase (26 to 149 days after disease onset), and reconvalescent-phase (150 to 499 days after disease onset) BMD samples, compared to samples from disease controls (culture-proven Lyme borreliosis [LB] patients), cross-reactive controls with other infectious diseases (ID; patients with serologically proven leptospirosis, syphilis, CMV, EBV, or HSV I/II infection or culture-proven H. influenzae bacteremia), healthy blood donors from regions (in Russia and The Netherlands) where *Ixodes* ticks are endemic, and healthy controls from regions (in Russia and Norway) where the ticks are not endemic. *B*., *Borrelia*; BMD, Borrelia miyamotoi disease; CMV, cytomegalovirus; E, endemic; EBV, Epstein-Barr virus; GlpQ, glycerophosphodiester phosphodiesterase; HBD, healthy blood donors; HSV, herpes simplex virus; Ig, immunoglobulin; ID, infectious diseases; LB, Lyme borreliosis; NE, nonendemic; Reconv., reconvalescent; Vlp, variable large protein; Vsp, variable small protein.

Antibody responses were measured by protein array in all available sera from patients with BMD and cross-reactive and healthy controls (Fig. 1). Significantly increased concentrations of IgM and IgG antibodies against GlpQ, all Vmps, and flagellin were observed in sera from acute-phase, early-convalescent-phase, late-convalescent-phase, and reconvalescent-phase patients with BMD compared to controls (see Appendix S4 in the supplemental material). Not unexpectedly, especially for Vlp-18 and flagellin, and to a lesser extent Vlp-15/16, a high reactivity was observed in Lyme borreliosis disease controls. The median IgM concentration peaked from 0 to 4 to 5 to 25 days, increasing 16-, 6-, and 5-fold against GlpQ, any Vmp, and flagellin, respectively. The median IgG concentration peaked from 0 to 4 to 26 to 149 days, increasing 154-, 56-, and 5-fold against GlpQ, any Vmp, and flagellin, respectively. Furthermore, the difference in antibody concentration was most pronounced from 5 to 149 DOD for IgM and 25 to 499 DOD for IgG.

### Threshold for reactivity for individual antigens by ROC analysis.

Receiver operating characteristic (ROC) curves were generated using data obtained with sera from BMD patients, categorized in seven time windows (0 to 4, 5 to 11, 12 to 25, 26 to 65, 66 to 149, 150 to 230, and 231 to 499 DOD) (17). Sera from the first six time windows were used for IgM (*n* = 135) and those from the last six time windows for IgG (*n* = 128) analysis. Sera from Russian controls from regions where *Ixodes* was endemic and nonendemic, as well as Norwegian healthy controls from regions where it is nonendemic, were used as negative controls (*n* = 280). The ROC curves for the individual antigens for IgM and IgG, with their corresponding areas under the curves (AUCs), are shown in Appendixes S5 and S6, respectively. Whereas AUCs in the first time window were above 0.6 for IgM (0 to 4 DOD) and 0.7 for IgG (5 to 11 DOD), AUCs were greater than 0.8 in all consecutive time windows for both IgM and IgG. We aimed for a binary discrimination threshold to reach a specificity above 95% for all antigens for both IgM and IgG. Based thereon, but also striving for uniformity, a cutoff of 5 μg/mL was selected for all individual antigens, accepting a 93% specificity for flagellin.

### Diagnostic accuracy of individual and combination of antigens.

To determine the diagnostic accuracy of the individual as well as combinations of antigens, sera from BMD patients were used to assess sensitivity, and sera from all available control groups (1,095 individuals) were used to determine specificity (Table 1). Control groups were categorized into four strata: (i) 71 Lyme borreliosis patients, (ii) 144 patients with other infectious diseases, (iii) 697 healthy individuals from regions of *Ixodes* endemicity, and (iv) 183 healthy individuals from regions of nonendemicity.

**TABLE 1:** Sensitivity and specificity of the protein array in BMD patients and control groups^*a*^

Ig	Antibody	BMD patients		LB		ID		HBD-E		HBD-NE		All controls	
		Sensitivity		Specificity		Specificity		Specificity		Specificity		Specificity	
		%	95% CI	%	95% CI	%	95% CI	%	95% CI	%	95% CI	%	95% CI
IgM	GIpQ	75.9	65.4–84.0	97.2	90.2–99.7	98.6	95.1–99.8	97.1	95.6–98.2	98.9	96.1–99.9	97.6	96.5–98.4
	Any Vmp	78.3	68.0–86.0	77.5	66.0–86.5	88.9	82.6–93.5	79.5	76.3–82.4	95.1	90.9–97.7	83.2	80.9–85.4
	Flagellin	66.3	55.3–75.7	60.6	48.3–72.0	94.4	89.4–97.6	88.7	86.1–90.9	97.3	93.7–99.1	89.0	87.0–90.8
	GlpQ or any Vmp	89.2	80.3–94.3	76.1	64.5–85.4	87.5	81.0–92.4	77.3	74.0–80.4	95.1	90.9–97.7	81.6	79.1–83.8
	GlpQ and any Vmp	57.8****	46.8–68.1	100.0	95.0–100.0	100.0	97.5–100.0	99.3****	98.3–99.8	98.9	96.1–99.9	99.4****	98.7–99.7
	GlpQ and (any Vmp or flagellin)	68.7^^,##^	57.8–77.8	100.0	95.0–100.0	100.0	97.5–100.0	99.0^^^	97.9–99.6	98.9	96.1–99.9	99.2^^^^	98.5–99.6
IgG	GlpQ	69.9	59.0–78.9	97.2	90.2–99.7	97.2	93.0–99.2	96.0	94.3–97.3	98.4	95.3–99.7	96.6	95.4–97.6
	Any Vmp	79.5	69.3–87.0	70.4	58.4–80.7	95.8	91.2–98.5	84.4	81.5–87.0	95.1	90.9–97.7	86.8	84.6–88.7
	Flagellin	37.3	27.5–48.4	56.3	44.1–68.1	91.7	85.9–95.6	90.4	88.0–92.5	92.3	87.5–95.8	88.7	86.7–90.5
	GlpQ or any Vmp	90.4	81.7–95.2	69.0	56.9–79.5	93.1	87.6–96.6	81.5	78.4–84.3	93.4	88.8–96.6	84.2	81.9–86.3
	GlpQ and any Vmp	56.6***	45.7–67.0	98.6	92.4–100.0	100.0	97.5–100.0	98.9****	97.8–99.5	100.0	98.0–100.0	99.2****	98.5–99.6
	GlpQ and (any Vmp or flagellin)	61.4^	50.4–71.4	97.2	90.2–99.7	99.3	96.2–100.0	98.4^^^^	97.2–99.2	100.0	98.0–100.0	98.7^^^^	97.9–99.3
IgM or IgG	GlpQ	88.0	78.9–93.5	94.4	86.2–98.4	95.8	91.2–98.5	93.1	91.0–94.9	97.3	93.7–99.1	94.2	92.7–95.6
	Any Vmp	94.0	86.2–97.5	62.0	49.7–73.2	86.1	79.4–91.3	68.9	65.3–72.3	90.2	84.9–94.1	74.2	71.6–76.8
	Flagellin	79.5	69.3–87.0	40.8	29.3–53.2	86.8	80.2–91.9	80.2	77.1–83.1	90.2	84.9–94.1	80.2	77.7–82.5
	GlpQ or any Vmp	97.6	90.7–99.4	60.6	48.3–72.0	81.9	74.7–87.9	65.6	61.9–69.1	88.5	83.0–92.8	71.2	68.5–73.9
	GlpQ and any Vmp	79.5*	69.3–87.0	98.6	92.4–100.0	100.0*	97.5–100.0	98.1****	96.8–99.0	98.9	96.1–99.9	98.5****	97.6–99.2
	GlpQ and (any Vmp or flagellin)	84.3	74.7–90.8	97.2	90.2–99.7	99.3	96.2–100.0	97.4^^^^	96.0–98.5	98.9	96.1–99.9	97.9^^^^^,#^	96.9–98.7
IgM and IgG	GlpQ	44.6	34.1–55.5	100.0	94.9–100.0	100.0	96.2–100.0	100.0	99.5–100.0	100.0	98.0–100.0	100.0	99.7–100.0
	Any Vmp	59.0	48.0–69.2	85.9	75.6–93.0	98.6	95.1–99.8	95.0	93.1–96.5	100.0	98.0–100.0	95.7	94.3–96.8
	Flagellin	24.1	16.0–34.6	94.4	86.2–98.4	100.0	96.2–100.0	99.9	99.2–100.0	100.0	98.0–100.0	97.5	96.4–98.4
	GlpQ or any Vmp	75.9	65.4–84.0	84.5	74.0–92.0	98.6	95.1–99.8	93.3	91.1–95.0	100.0	98.0–100.0	94.5	93.0–95.8
	GlpQ and any Vmp	26.5****	8.0–37.2	100.0	94.9–100.0	100.0	96.2–100.0	100.0	99.5–100.0	100.0	98.0–100.0	100.0	99.7–100.0
	GlpQ and (any Vmp or flagellin)	37.3^^,##^	27.5–48.4	100.0	94.9–100.0	100.0	96.2–100.0	100.0	99.5–100.0	100.0	98.0–100.0	100.0	99.7–100.0

a For combinations of immunoglobulins/antigens, “or” indicates that either immunoglobulin/antigen tested positive, and “and” indicates that both immunoglobulins/antigens tested positive. The four control groups comprise disease controls (culture-proven Lyme borreliosis patients), cross-reactive controls with other infectious diseases (patients with serologically proven leptospirosis, syphilis, CMV, EBV, or HSV I/II infection or culture-proven H. influenzae bacteremia), healthy controls from regions (in Russia and The Netherlands) where *Ixodes* ticks are endemic, and healthy controls from regions (in Russia and Norway) where they are not. Sensitivity within BMD patients and specificity within the control groups are compared. Significance was calculated by exact McNemar test by comparing different diagnostic algorithms: one symbol, *P* < 0.05; two symbols, *P* < 0.01; three symbols, *P* < 0.001; four symbols, *P* < 0.0001. *, comparison between GlpQ and the algorithm “GlpQ and any Vmp”; ^, comparison between GlpQ and the algorithm “GlpQ and (any Vmp or flagellin)”; #, comparison between the algorithms “GlpQ and any Vmp” and “GlpQ and (any Vmp or flagellin).” BMD, Borrelia miyamotoi disease; CI, confidence interval; E, endemic; GlpQ, glycerophosphodiester phosphodiesterase; HBD, healthy blood donors; Ig, immunoglobulin; ID, infectious diseases; LB, Lyme borreliosis; NE, nonendemic; Reconv., reconvalescent; Vmp, variable major protein.

The sensitivities of IgM/IgG against the individual antigens were 88.0% (95% confidence interval [CI], 78.9 to 93.5), 94.0% (95% CI, 86.2 to 97.5) and 79.5% (95% CI, 69.3 to 87.0) for GlpQ, any Vmp, and flagellin, respectively. The specificities of all control groups combined were 94.2% (95% CI, 92.7 to 95.6) for GlpQ, 74.2% (95% CI, 71.6 to 76.8) for any Vmp, and 80.2% (95% CI, 77.7 to 82.5) for flagellin. The reactivity varied throughout the four control groups, with lowest specificity in the healthy controls from regions of *Ixodes* endemicity for GlpQ. In contrast, both any Vmp and flagellin showed the lowest specificity for IgM/IgG in the LB disease control group, 62.0% (95% CI, 49.7 to 73.2) and 40.8% (95% CI, 29.3 to 53.2), respectively. Healthy individuals with a residence in *Ixodes* endemic areas in Russia or The Netherlands showed IgM/IgG specificities of 93.1% (95% CI, 91.0 to 94.9) for GlpQ, 68.9% (95% CI, 65.3 to 72.3) for any Vmp, and 80.2% (95% CI, 77.1 to 83.1) for flagellin, whereas healthy individuals from areas of *Ixodes* nonendemicity in Russia or Norway showed IgM/IgG specificities of 97.3% (95% CI, 93.7 to 99.1) for GlpQ and 90.2% (95% CI, 84.9 to 94.1) for both any Vmp and flagellin.

**FIG 2:**
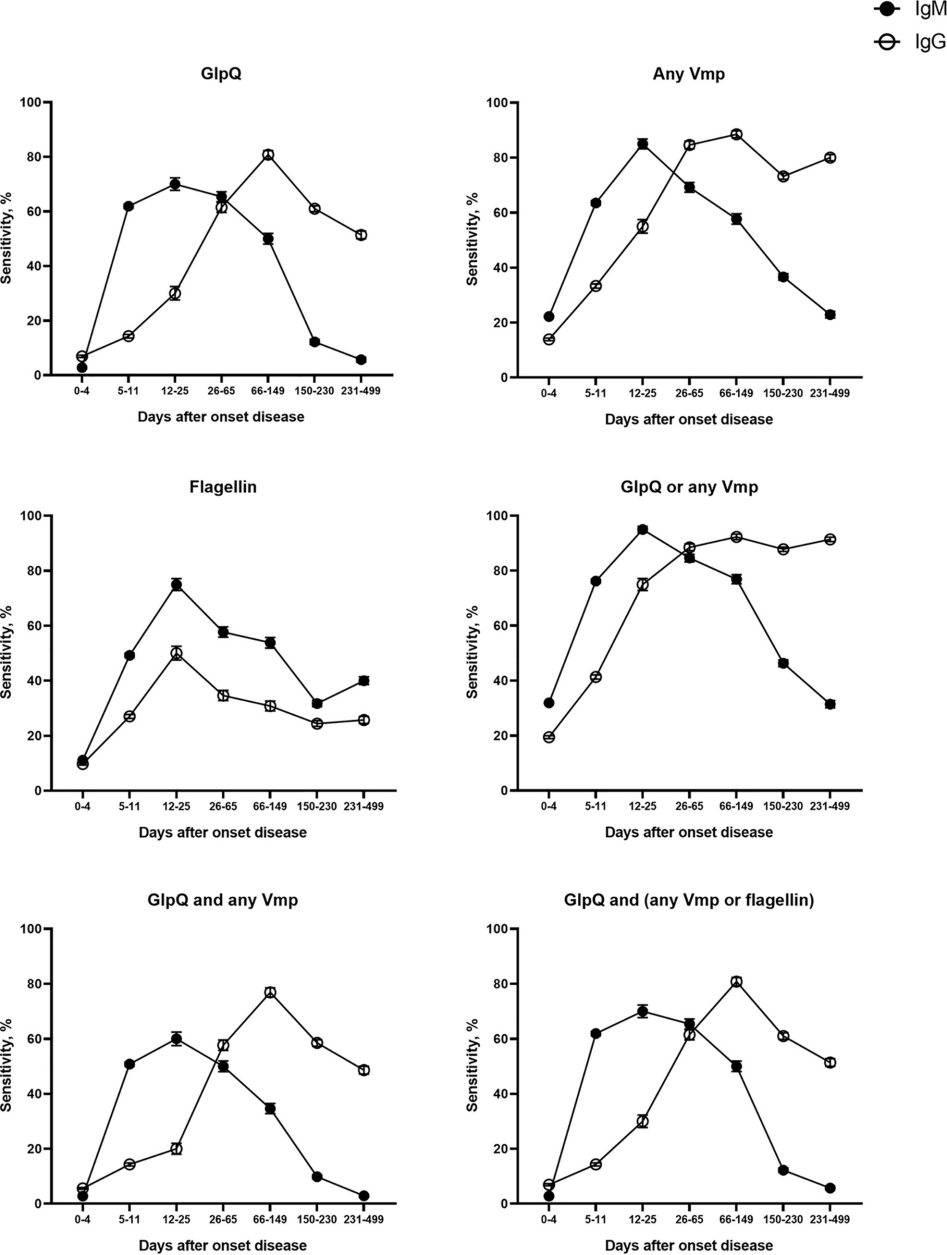
Sensitivity of individual antigens and combinations of antigens in the protein array for BMD patients over time. Sensitivity with standard error of the mean for IgM and IgG is given for individual antigens and combinations of antigens. The sensitivity is shown for BMD patients for each time window. Cutoffs for both IgM and IgG were set at 5 μg/mL with a specificity of >95% for IgM and a specificity of >93% for IgG. Combinations of antigens are shown with either “or” or “and.” “Or” indicates that either antigen-specific-antibody level was increased; “and” indicates that both antigen-specific-antibody levels were increased. BMD, Borrelia miyamotoi disease; GlpQ, glycerophosphodiester phosphodiesterase; Ig, immunoglobulin; Vmp, variable major protein.

It is generally accepted that the diagnostic accuracy, especially the specificity, improves by combining the individual antigens as a multiple-tier test (MTT). The dynamics of individual antigens and MTT sensitivity over time is shown in Fig. 2 and Appendix S7. The previously published optimal diagnostic algorithm, defining seroreactivity for BMD as reactivity against GlpQ together with any Vmp, resulted in this array, with sensitivities of 57.8% (95% CI, 46.8 to 68.1) for IgM, 56.6% (45.7 to 67.0) for IgG, and 79.5% (69.3 to 87.0) for IgM/IgG and specificities of 99.4% (95% CI, 98.7 to 99.7), 99.2% (95% CI, 98.5 to 99.6) and 98.5% (95% CI, 97.6 to 99.2) for IgM, IgG, and IgM/IgG, respectively. Whereas the specificities were significantly higher, this came at the cost of significantly lower sensitivities, compared to GlpQ alone. Interestingly, by stepwise logistic regression of all possible combinations of individual antigens, we identified an alternative advantageous algorithm, defined as reactivity against GlpQ in combination with any Vmp or flagellin (referred to here as MTT-GVF). MTT-GVF resulted in sensitivities of 68.7% (95% CI, 57.8 to 77.8) for IgM, 61.4% (95% CI, 50.4 to 71.4) for IgG, and 84.3% (95% CI, 74.7 to 90.8) for IgM/IgG and specificities of 99.2% (95% CI, 98.5 to 99.6), 98.7% (95% CI, 97.9 to 99.3), and 97.9% (95% CI, 96.9 to 98.7) for IgM, IgG, and IgM/IgG, respectively. It is noteworthy that these sensitivities were comparable to that for GlpQ alone, and the specificity was significantly higher.

## DISCUSSION

In the current study, to our knowledge the largest of its kind, we extensively investigated a novel Borrelia miyamotoi protein array as a serological tool to diagnose BMD, making use of an unique Russian cohort of BMD patients, various cross-reactive patients, and healthy controls from regions where *Ixodes* is endemic and those where it is not.

As hypothesized, we showed that the array had a markedly improved diagnostic value over the use of the conventional one-tier GlpQ marker and the recently proposed MTT algorithm defining positivity as reactivity to both GlpQ and any Vmp. Despite a single previous publication suggesting that GlpQ is unfit as a diagnostic antigen to detect anti-B. miyamotoi antibodies (27), our study confirmed that as a single antigen, GlpQ remains the main discriminatory marker in BMD. This is well in line with multiple publications from our own group and others (17–19). Interestingly, addition of the Vmp markers by implementing the previous diagnostic MTT algorithm significantly improved specificity. Unfortunately, but as to be expected, this increase did come at the cost of a slight yet significant decrease in sensitivity in IgM, IgG, and IgM/IgG. This could be circumvented by replacing the previously published MTT algorithm by a novel algorithm defining positivity as reactivity to GlpQ and either any Vmp or flagellin. This novel algorithm, designated MTT-GVF, resulted in sensitivities for IgM, IgG and IgM/IgG comparable to, and in a specificity significantly higher than, that of GlpQ alone. We therefore propose that the current multiantigen protein array could be implemented in daily diagnostic routines and could be used for epidemiological studies assessing exposure to B. miyamotoi.

A limitation of our study was that we were bound by a case-control design using convenient samples from clinical practice, rather than consecutive samples collected through a cohort design. Such a one-gate design is preferred for all diagnostic test accuracy studies; however, it is practically impossible to design for BMD diagnostic studies. It was also not possible to perform an independent assessment of the array in both a discovery and validation case cohort, due to the absence of another available BMD cohort. However, by the use of this unique and large BMD cohort, together with a vast number and variety of disease and healthy controls from both Russia and Europe, we did acquire an unprecedented population in which we obtained adequate power to reliably describe the diagnostic accuracy of the array. Although the recombinant Vmp antigens used in the array were obtained from an American and Japanese isolate to ascertain reactivity in European and Russian samples, Vmps from the different subgroups were chosen covering a broad spectrum of diversity. Moreover, a high level of cross-reactivity is to be expected between Vmps from different geographical regions (28). Additionally, sequences of *glpQ* and the flagellin gene were highly conserved within relapsing fever *Borrelia* species and within all *Borrelia* species, respectively. Finally, it should be mentioned that our novel diagnostic array and algorithm can be used to distinguish between B. miyamotoi and B. burgdorferi sensu lato in regions where no other relapsing fever *Borrelia* are prevalent. Although this was not investigated, our array most likely does not discriminate between antibodies in response to infection with B. miyamotoi and other relapsing fever *Borrelia* species.

### Conclusions.

The multiantigen B. miyamotoi protein array and the MTT-GVF algorithm aid in the serodiagnosis of BMD. The array is standardized and high throughput, and it is based on a sophisticated technique which could greatly facilitate implementation into routine diagnostics. The proposed diagnostic algorithm has an excellent specificity in cross-reactive and healthy controls with a more-than-adequate sensitivity in BMD patients. Further research should focus on the validation of the array with an independent BMD cohort, preferably from another geographical region, and sera from patients infected with other relapsing fever *Borrelia* species. In addition, genetic variants of the known antigens, as well as novel antigens, such as the recently identified surface lipoprotein BmaA (29), could be investigated. With the current protein array, we hope to aid in a better understanding of the burden of infection and disease caused by the emerging hard-tick-borne pathogen B. miyamotoi.

## MATERIALS AND METHODS

### BMD cases and controls.

To validate the protein array as a diagnostic test, we studied confirmed BMD cases and potentially cross-reactive and various healthy controls (Table 2). Sera from cases comprise 283 samples from 83 patients enrolled at two study locations; 28 cases in Izhevsk (European Russia) in 2014 to 2016, and 55 cases in Yekaterinburg (Asian Russia) in 2015 to 2016. Their clinical manifestations were consistent with BMD (15). Infection was demonstrated by B. miyamotoi-specific qPCR (10, 30, 31). An alternative diagnosis of, or concomitant coinfection with, B. burgdorferi sensu lato was ruled out (10, 15, 30, 31). Sera were collected at admission, during treatment, and at follow-up and were categorized by time of sampling after disease onset, in two manners. The first division constituted four strata (acute, early convalescent, late convalescent, and reconvalescent phases) drawn at 0 to 4, 5 to 25, 26 to 149, and 150 to 499 DOD, respectively. Subsequently, a more elaborate grouping was made using seven successive time windows, including 72 samples from 0 to 4, 63 samples from 5 to 11, 20 samples from 12 to 25, 26 samples from 26 to 65, 26 samples from 66 to 149, 41 samples from 150 to 230 and 35 samples from 231 to 499 DOD.

**TABLE 2:** Characteristics of cohorts used in this study^*a*^

Group	Description of cohort					Time points (days), *n*	
	Geography	Inclusion year(s)	No. of participants	No. of samples	Selection/diagnosis	A	B
Cases							
Borrelia miyamotoi disease	Russia, Izhevsk and Yekaterinburg	2014–2016	83	Time point A, 241 Time point B, 283	Physicians confirmed clinical manifestations of tick-borne fever i.c.w. molecular confirmation B. miyamotoi infection.	Acute (0–4), 72 Early conv. (5–25), 66 Late conv. (26–149), 46 Reconv. (150–499), 57	Window 1 (0–4), 72 Window 2 (5–11), 63 Window 3 (12–25), 20 Window 4 (26–65), 26 Window 5 (66–149), 26 Window 6 (150–230), 41 Window 7 (231–499), 35
Cross-reactive controls							
Lyme borreliosis	Netherlands, nationwide	1986–2018	71	132	Physicians confirmed skin manifestation, all i.c.w. culture confirmation and molecular affirmation of Lyme borreliosis	Acute (0–41), 69 Early conv. (42–83), 38 Late conv. (≥84), 25	NA
Other infectious disease	Russia, Moscow	2013	93	93	Physician confirmed clinical manifestations and serological confirmed leptospirosis, syphilis, CMV, EBV, or HSV-I/II.	Acute (NA), 93	NA
	Netherlands, Amsterdam	2013–2014	51	51	Physician confirmed clinical manifestations and serological confirmed leptospirosis, syphilis, CMV, EBV, or culture proven Haemophilus influenzae bacteremia.	Acute (NA), 51	NA
Healthy controls							
HBD, region of endemicity	Russia, Izhevsk	2014–2016	97	97	Healthy blood donors from the same *Ixodes* endemic region as the BMD cases	Random (NA), 97	NA
	Netherlands, nationwide	2018–2019	600	600	Healthy blood donors randomly selected with an even distribution of age, sex, and geographical residency in the endemic for *Ixodes*	Random (NA), 600	NA
HBD, region of nonendemicity	Russia, Volgograd	1999	83	83	Healthy blood donors from a region of *Ixodes* nonendemicity in Russia	Random (NA), 83	NA
	Norway, northern region	2012	100	100	Healthy blood donors who never observed tick bites from a region of *Ixodes* nonendemicity in northern Norway	Random (NA), 100	NA

a BMD, Borrelia miyamotoi disease; CMV, cytomegalovirus; EBV, Epstein-Barr virus; HBD, healthy blood donors; HSV, herpes simplex virus; i.c.w., in combination with; *n*, number of samples per time window; NA, not applicable; Reconv., reconvalescent.

As disease controls, 132 serum samples from 71 Dutch patients with culture-proven LB were included, propagated from physician-confirmed solitary erythema migrans (EM; 58 patients), in combination with other disseminated manifestations or acrodermatitis chronica atrophicans (13 patients). They comprised 26 acute-phase (0 to 6 weeks), 24 early-convalescent-phase (6 to 12 weeks), and 77 late-convalescent-phase (>12 weeks) samples and 5 samples from undetermined time points (drawn at first presentation of EM with unknown DOD), collected in 1986 to 2018. In addition, as potential cross-reactive controls, we included sera from patients with serologically confirmed infectious diseases from both Russia (93 samples) and The Netherlands (51 samples), collected in 2013 and in 2013 to 2014, respectively. This group consists of serum samples from patients with IgM-confirmed cytomegalovirus infection (40 patients), Epstein-Barr virus infection (24 patients), or herpes simplex virus I/II infection (10 patients), IgM/IgG-confirmed active leptospirosis (15 patients) or VDRL- and anti-Treponema pallidum ELISA-positive syphilis (59 patients), or culture proven Haemophilus influenzae bacteremia (3 patients).

Finally, we included various healthy control groups from regions where *Ixodes* ticks are endemic (697 individuals) and regions where they are not (183 individuals). Sera from healthy controls from regions of endemicity consisted of 97 Russian donor samples collected in Izhevsk in 2014 to 2016 and 600 Dutch donor samples selected to provide an even spread of sex, age category, and geographical location across The Netherlands, collected in 2018 to 2019. The samples from healthy controls in regions of nonendemicity included 83 samples collected in Volgograd, Russia, in 1999 and 100 samples acquired from patients who never observed tick bites collected in northern Norway in 2012.

### Recombinant protein generation.

The array includes five B. miyamotoi specific antigens obtained from the American tick isolate LB-2001 (32) and the Japanese tick isolate Ht-31 (3), consisting of GlpQ and four Vmps. GlpQ (ON951671) and a single Vsp (Vsp-1; ON931606) were derived from LB-2001. Three Vlps, one each from the subgroup α family (Vlp-18; ON931609), γ family (Vlp-5; ON931607), and δ family (Vlp-15/16; ON931608), were generated from Ht-31. In addition, the assay contains several B. burgdorferi sensu lato-specific antigens, which are not further specified in this paper, with the exception of flagellin B obtained from the B. afzelii strain pKo (ON951672) and B. bavariensis strain Bpi (ON951673) (33, 34). The genes encoding the recombinant full-size proteins were either made by gene amplification or assembled *de novo*. Accession numbers, cloning primers, gene sequences, and the protocol of synthesis can be found in Appendixes S1 and S2 (22, 32–36).

### Protein array.

The protein array preparation, procedure, and readouts were performed as described in detail in Appendix S3 (37–39). Briefly, antigens were spotted in triplicate on aldehyde-activated glass slides, including a negative printing control (phosphate-buffered saline), positive printing control and calibration marker (purified IgM and IgG series), and array boarder markers (cystine-3 and cystine-5 *N*-hydroxysuccinimide ester-labeled bovine serum albumin). Serum samples were diluted 1:10, and selected human sera were pooled to generated negative and positive serum controls. Fluorescence readout was performed with a MArS laser scanner (Ditabis, Germany). Images were quantified using SpotScout. Finally, readouts were corrected for intratest variation and background signal and quantified in micrograms per milliliter.

### Ethics statement.

The study was conducted according to the principles of the Declaration of Helsinki and in conformity with institutional regulations and guidelines. Written informed consent was obtained from the Russian BMD patients and controls in accordance with and with approval from the institutional review boards of the Republican Hospital of Infectious Diseases (Udmurt Republic, Russia), the medical association Novaya Bolnitsa (Yekaterinburg, Russia), and the Council on Bioethics of the Izhevsk State Medical Academy (minutes no. 17 of 24 December 2012) and from the Norwegian controls by the Regional Committee for Medical and Health Ethics (REC North no. 2011/2575). The Dutch healthy controls provided explicit written consent for the use of their deidentified leftover materials for research purposes. The additional control cohorts comprise deidentified patient materials left over from standard clinical practice; therefore, the Dutch Medical Research Involving Human Subjects Act does not apply to these samples, and no informed consent was needed.

### Statistical analysis.

Analyses were performed using STATA15 (StataCorp LLC, College Station, TX, USA) and GraphPad Prism 7.0.2 (GraphPad Prism Software Inc., La Jolla, CA, USA). We stratified the study cohorts in three groups: (i) proven BMD patients, (ii) potentially cross-reactive controls, and (iii) healthy controls. The samples of the first group were subsequently divided into different time windows, in which only a single sample from a unique participant was selected per subgroup. The seroreactivity against the most immunogenic flagellin genetic variant was chosen for analysis. ROC analyses were performed to determine the cutoff for reactivity per antigen. Numbers of positives and percentages were pooled, depending on the study stratum. Mann-Whitney U tests were used to compare groups, and the McNemar test was used within a group. A *P* value below 0.05 was considered statistically significant. Diagnostic algorithm analyses were performed by stepwise logistic regression.
