# Supplementary material for: Development and Validation of a Protein Array for Detection of Antibodies against the Tick-Borne Pathogen Borrelia miyamotoi
Source: Microbiol Spectr. 2022 Oct 31;10(6):e02036-22. doi: 10.1128/spectrum.02036-22 (PMC9769530; doi:10.1128/spectrum.02036-22)
Supplement: Supplemental file 1 — Supplemental material. Download spectrum.02036-22-s0001.pdf, PDF file, 0.5 MB [file spectrum.02036-22-s0001.pdf]

Table of content

Title		Page
Table of content		1
Supplemental appendix 1	Characteristics and protocol for recombinant protein synthesis	2
Supplemental appendix 2	Properties of recombinant proteins solutions	8
Supplemental appendix 3		9
Supplemental appendix 4	Antibody levels in BMD patients and control groups	10
Supplemental appendix 5	ROC curves for IgM and IgG against individual B. miyamotoi antigens in BMD patients and control groups	12
Supplemental appendix 6	Diagnostic power of the measurements of specific IgM- and IgG-antibodies represented as Area Under the Curve of Receiver Operating Characteristics	13
Supplemental appendix 7	Sensitivity and specificity of individual and combination of antigens of the protein array in BMD patients over time	14
References		16

Supplemental appendix 1. Characteristics and protocol for recombinant protein synthesis

Gene	*Borrelia* species, Strain name	NCBI gene reference	Oligo-nucleotide	Oligonucleotide sequence	Restriction enzymes, vector
GlpQ	*B. miyamotoi*, LB-2001	ON951671	glpQ-F	cc atg gaa atg ggt tca aac aaa aag tca cca ttg atc ata gct cac ag	NcoI/XhoI, pET16
			glpQ-R	ctc gag ttt ttt tat gaa gtt cat tac tgt gtc ag	
Vsp-1	*B. miyamotoi*, LB-2001	ON931606	Vsp1-A	tactgaattcgcatatgaagaaacgc	NdeI/NotI, pGD
			Vsp1-1	gatcagaaacagggtcatgataattgcgctcagagttttgcgtttcttcatatgcgaattc	
			Vsp1-2	aattatcatgaccctgtttctgatcattaacattgtgatgattagctgcggcagcggtggc	
			Vsp1-3	accacggtgccatccgccttagccgcctggccctctttcggagctgggccaccgctgccgc	
			Vsp1-4	cggatggcaccgtggtggatctggttaaagtatcgaagaagattaaagatgcggtggaatt	
			Vsp1-5	ttttcaccagggtttccacttctttcacattcgccgcaaattccaccgcatctttaatctt	
			Vsp1-6	aagtggaaaccctggtgaaaagcattgatgaactggcgaaggccattggcaaaaagataaa	
			Vsp1-7	ccatttttaccgctttcggtatcaaactggccatcgctttttatctttttgccaatggcct	
			Vsp1-8	taccgaaagcggtaaaaatggcagcctgctggcgggtgcacagagcattatgctggcagtg	
			Vsp1-9	ctaatgccttctttattgtccagctgacccagtttcgctttcactgccagcataatgctct	
			Vsp1-10	gctggacaataaagaaggcattagcaccgagctgaaacagaaagtgaccgatagcaagacc	
			Vsp1-11	tatggttgtctttcagtttggtcagaaaggtttcggttttggtcttgctatcggtcacttt	
			Vsp1-12	gaccaaactgaaagacaaccatagcgatctgggcaagaatgaagcgaccgatgcgcacgcg	
			Vsp1-13	gtgcctttgtccttggtgccggtatcggtaatatcaatcgcgcttttcgcgtgcgcatcgg	
			Vsp1-14	gcaccaaggacaaaggcaccagcgaactgatagcgctgaataccagtattaatgcgttact	
			Vsp1-15	ttaatcgccgcttcgacttcatcattcgcggtctccagtaacgcattaatactggtattca	
			Vsp1-16	aagtcgaagcggcgattaaggcgctgataaatccgagcaaagcgttgaccgctggccaaag	
			Vsp1-B	agttctcgagttatgcggccgcgctgctttggccagcggtcaa	
			Vsp1-A1	tactgaattcgcatatgggcccagctccgaaagagggccaggcggctaaggcggatggc	
Vlp-5	*B. miyamotoi*, Ht-31	ON931607	Vlp5-A	tactgaattcgcatatgcttggcgtggtgagctgcaattcca	NdeI/NotI, pGD
			Vlp5-1	caggaacttgctctgcgggctttcttccgccacagtgccgctggaattgcagctcaccac	
			Vlp5-2	ccgcagagcaagttcctgaaaagcgtgattagcctgggcaacaatttccttaacgtgttt	

			Vlp5-3	atcccagcacgcctcccaccatatcgccaaagctagtaaacacgttaaggaaattgttgc	
			Vlp5-4	ggaggcgtgctgggattcaatagcaataccaaaaagagcgatgtaggcgtctatttcaag	
			Vlp5-5	aggctggttttggtgccttccacggtatcatgcactttcttgaaatagacgcctacatcg	
			Vlp5-6	ggcaccaaaaccagcctggagaagatagttgcagatatgaaaaccgaagggaatccgaac	
			Vlp5-7	tatcgatcagtttttcacagcggtgtcggtcgcctccgcgttcggattcccttcggttt	
			Vlp5-8	cgctgtgaaaaaactgatcgataataccctgagcaaaatcattgccggtgcaaagaccgc	
			Vlp5-9	cgttccccagcagatcattaccttcaatgccaatagcttcgctcgcggtctttgcaccgg	
			Vlp5-10	taatgatctgctggggaacgtcgcggaccaaaatgacggtgccgtgggcatcgaagttga	
			Vlp5-11	agcaccacatccacaatgcttttaatgcctttcaccagcttgtcaacttcgatgcccacg	
			Vlp5-12	gcattgtggatgtggtgctgaaggagggtaaacatgatgcgggggatgataaaaaggcct	
			Vlp5-13	tcacccgcattaccccggtacgcgcggtgctgccgtctgaggcctttttatcatccccc	
			Vlp5-14	gggggtaatgcgggtgacggcgaggcgggcaaactgtttgcaagcgcgaatgcaggcacg	
			Vlp5-15	accgccttcgccgcgtctgccgcagatttctttgcgttctccgccgtgcctgcattcgcg	
			Vlp5-16	gcggcgaaggcggtcggcagcgtgaccggagccgatattctgcaggcgattgtcaaggat	
			Vlp5-17	accgctgcattattgctcgccagtttaatcgcttcaccattatccttgacaatcgcctgc	
			Vlp5-18	cgagcaataatgcagcggttgcggcggtggataccaacaaaaaagatggcaccatagctg	
			Vlp5-19	atttgccccctttcgccatcgcacgcagcgccatgccgccagctatggtgccatcttttt	
			Vlp5-20	ggcgaaagggggcaaattcgctggcccgtcagatgcggcgagcgcccgacgcgaagaaaat	
			Vlp5-21	tccagcgcttttgtcacggccgataccgccactccctctacgattttcttcgcgtcggcg	
			Vlp5-22	cgtgacaaaagcgctggacaccctgaccattgcgattcgtaaaaccattgatctgggcct	
			Vlp5-23	tcggacgcgttaatcttcattgcttcctgcacttctttcaggcccagatcaatggtttta	
			Vlp5-24	aatgaagattaacgcgtccgatactccggtgatttcggataagaagacctcagaggcgaa	
			Vlp5-B	agttctcgagttatgcggccgcttccgatttcgcctctgaggtcttctta	
Vlp-15/16	*B. miyamotoi*, Ht-31	ON931608	Vlp15/16-A	tactgaattcgcatatgagcaaacgtaagaccctgagtgcgatcattatgaccct	NdeI/NotI, pGD
			Vlp15/16-1	tcttccccgccaccattgttacagcctatgatcaggaacagggtcataatgatcgcactc	
			Vlp15/16-2	aatggtggcggggaagatccgcagaaagtgtttttgacaagcattgctaatctgggaaaa	
			Vlp15/16-3	accatatcgccaaaggtcacaaacacatccaggaagccttttcccagattagcaatgctt	
			Vlp15/16-4	gtgacctttggcgatatggtgactggggcgtttggcattaaagccgagaccaagaaaagc	
			Vlp15/16-5	tccatggtttccgcgatgctggtaaaatactggcccacttcgcttttcttggtctcggct	

			Vlp15/16-6	catcgcggaaaccatggaatctgtgaagaaaaaactgcaatctgaagtggcgaaaaacgg	
			Vlp15/16-7	taatgaatttatccaccacggttttcactttttcatagtttccgtttttcgccacttcag	
			Vlp15/16-8	aaaaccgtggtggataaattcattactgaaaccttagacaaaattgcgtccggcgcaaaa	
			Vlp15/16-9	agcgttgccgatcgccgcatcgccattcgcccccttttgcagcctcttttgcgccggacgc	
			Vlp15/16-10	ggcgatcggcaacgctgttaagaacgaagatgctgccccaggggaagtagcgtccgtaaa	
			Vlp15/16-11	gcaccacgcccacgatttccttgatgcctttcaccagcgcgtttacggacgctacttccc	
			Vlp15/16-12	tcgtgggcgtggtgctgaaaaaaggcgaaggagatagcgaagcgaccaaaacggcggaca	
			Vlp15/16-13	ttagcacctttgcccagcagtttccctacgctcttcttctcggtgtccgccgttttggtc	
			Vlp15/16-14	gctgggcaaaggtgctaatgatggcaccgaaacccaggccgcagccgcgtccgcgagcat	
			Vlp15/16-15	gatttagcgattgcctgcagaatatccacgccgctcaccgcgccaatgctcgcggacgcg	
			Vlp15/16-16	ctgcaggcaatcgctaaatcaggcgaggcagctaccggcgaaattaagattgaggaagca	
			Vlp15/16-17	tatcggccttggcagcagcaatttcagcggcattctttgcttcctcaatcttaatttcgc	
			Vlp15/16-18	tgctgccaaggccgatagcaaggatctggaaattgatagcgcgaagaaggatgcggtgat	
			Vlp15/16-19	taccatttttggccatcgcacgaagcgcaatacccccagcaatcaccgcatccttcttcg	
			Vlp15/16-20	tgcgatggccaaaaatggtaaatttgcggctaagaataatgaagacaagagcgcgcatag	
			Vlp15/16-21	acagggttttacctacggcgctggccgctacgccgttcacgctatgcgcgctcttgtctt	
			Vlp15/16-22	gccgtaggtaaaaccctgtctaccttgattattgcgattcgtaacacagtggattcgggc	
			Vlp15/16-23	tcttgcttcacggtagccagcacttcgttaatcgctttcaggcccgaatccactgtgtta	
			Vlp15/16-24	tggctaccgtgaagcaagaggataaaagcggggagagcaccaatgcggcagaggtcacga	
			Vlp15/16-B	agttctcgagttatgcggccgctttctgtgcagaagtcgtgacctctgccgc	
			Vlp15/16-A1	tactgaattcgcatatggaagatccgcagaaagtgtttttgacaagcattgctaatctg	
Vlp-18	*B. miyamotoi*, Ht-31	ON931609	Vlp18-A	tactgaattcgcatatggaaaaacgtagcgaacgtaa	NdeI/NotI, pGD
			Vlp18-1	atcatcagcagcacgatcatcataatgatcagaatgtttttacgttcgctacgtttttcc	
			Vlp18-2	gatgatcgtgctgctgatgattggttgcggccaacagactgaaggcaactcagcggcctct	
			Vlp18-3	cccacttccatcatcgcacccgacaggcccgagcccacgccgccagaggccgctgagttg	
			Vlp18-4	gtgcgatgatggaagtgggccgtagcgcggaaaatgtgttttatgcgtttatggaactgg	
			Vlp18-5	gtggtggtcttcgcagtgaagcctaacacatcgctcaccagttccataaacgcataaaac	
			Vlp18-6	cactgcgaagaccaccaccaagaaaaatgatgtgggcgtgtactttaacagcttaggcgt	
			Vlp18-7	ctacttcttccagctctttacttgcttcgcccagtttcacgcctaagctgttaaagtaca	

			Vlp18-8	caagtaaagagctggaagaagtagctaagaaagcggaaaccggcattgataagaatgata	
			Vlp18-9	tgacttcgacggcttctttgattaagttttttgagctatcattcttatcaatgccggttt	
			Vlp18-10	caaagaagccgtcgaagtcaccaaaaaagtgctggcgaccttaaaaggccatctggagag	
			Vlp18-11	cgtcccccaccagattggaatcgcccacctggcccaggctctccagatggccttttaagg	
			Vlp18-12	ccaatctggtgggggacgcggcgaccgatgataagggcgtgaccgctggcaccgacgcgc	
			Vlp18-13	tccgcaatatcgattatccccttcagggctttaaacgcccctttcagcgcgtcggtgcca	
			Vlp18-14	ggggataatcgatattgcggaaggcgcaggcgtggccaaaccgaaagctggctccaccgc	
			Vlp18-15	aggattttcgcgccatctttgttatccgcattgctcagcttcacggcggtggagccagct	
			Vlp18-16	aagatggcgcgaaaatcctggcaaccgacaataaagcgggcgtcaatgacgtgggcaaag	
			Vlp18-17	taaaatttcctccccgcttacgctcgcaagaatcactgccgctttgcccacgtcattgac	
			Vlp18-18	cgtaagcggggaggaaattttagcgagcattgtggaaagcaccgaaaataaggcggtcaa	
			Vlp18-19	ttccagcggggtggtagatgctgtcacattggtgctaattttgaccgccttattttcggt	
			Vlp18-20	ctaccaccccgctggaatttgcgttaggcggcaatggcgctcatctggcacaggatgcgg	
			Vlp18-21	aggctacgcagcgcaattccgccgctaaccgcgcttgccaacgccgcatcctgtgccaga	
			Vlp18-22	ttgcgctgcgtagcctggtgaaggatggcaaactggcgtcaggggctgcagatgggagcg	
			Vlp18-23	tgccgcggtaatccccactttctgcacttcctccttgccacccgcgctcccatctgcagc	
			Vlp18-24	ggggattaccgcggcaaataagctgctgggtgcggtcgaggatatcataaagaagaccgt	
			Vlp18-25	tcaatttttgtcttggctttctccagtatattcttgacggtcttctttatgatatcctcg	
			Vlp18-26	gagaaagccaagacaaaaattgatgaagcgcgtaatagcaaaaccgcggaagcggccgca	
			Vlp18-B	agttctcgagttatgcggccgcttccg	
			Vlp18-A1	tactgaattcgcatatgactgaaggcaactcagcggcctctggcggcgtgggctcgggc	
p41	*B. afzelii*,<br>pKo;<br>*B. bavariensis*,<br>Bpi	ON951672;<br>ON951673	p41-F	ttt ttt cat atg caa tat aac caa tg cac atg ttg tc	NdeI/KpnI,<br>pET16
			p41-R	ttt ttt ggt acc tta agc aag aga tgt att agc (ag)tc aac tgt	

Characteristics of recombinant full size protein generation by gene amplification (GlpQ and p41) and *de novo* synthesis (Vsp-1, Vlp-15/16, Vlp-18 and Vlp-5). The description includes the names of the genes, strains and isolates they were obtained from, the accession numbers, the oligo-nucleotide name and sequences, as well as the restriction enzymes and vectors used the recombinant protein production.

Abbreviations: B.: *Borrelia*, GlpQ: glycerophosphodiester phosphodiesterase, mg: milligram, ml: millilitre, p41: flagellin, Vlp: variable large protein, Vsp: variable small protein.

Protocol of recombinant protein generation

The array includes five *B. miyamotoi* specific antigens obtained from the American LB-2001 (genome CP006647) and Japanese HT31 (plasmids WP044003984.1, WP025444482, and WP025444235) tick isolates, consisting of GlpQ (ON951671) and four Vmps: a singular Vsp (Vsp-1, ON931606) and three Vlps (one from the subgroup α-family (Vlp-18, ON931609), γ-family (Vlp-5, ON931607), and δ-family (Vlp-15/16, ON931608), respectively). In addition the assay contains several *B. burgdorferi* s.l. specific antigens, which will no further be specified in this paper, with the exception of flagellin-B (ON951672 and ON951673) obtained from the *B. afzelii* strain pKo (genome CP002933) and *B. bavariensis* strain Bpi (genome CP000013). The recombinant full size proteins were either made by gene amplification or assembled *de novo*. Gene sequences from GlpQ and p41 were obtained by DNA extracts (DNeasy Blood & Tissue Kit, Qiagen, Hilden, Germany) from *Borrelia* cultures. PCR was performed with Phusion highfidelity mix (ThermoFisher Scientific, Waltham, USA) and carried out by denaturation at 94°C for 1 minute, followed by 25 cycles of 94°C for 30 seconds, 55°C for 40 seconds and 72°C for 50 seconds, followed by extension phase at 72°C for 10 minutes.

Genes encoding Vsp-1, Vlp-15/16, Vlp-18 and Vlp-5 proteins were synthesized *de novo*, using a two-step PCR as described previously (Dolgova *et al.* 2017). Amino acid alternations were made to optimize protein formation and reduce solubility. In Vsp-1 two base point mutations were substituted (N75→D75, D96→G96), and signal peptide detected by SignalP 4.1 (https://services.healthtech.dtu.dk/service.php?SignalP-4.1) were omitted in Vsp-1 (amino acid 1-28), Vlp-15/16 (amino acid 1-23) and Vlp-18 (amino acid 1-26). Primers were designed using DNAWorks (https://hpcwebapps.cit.nih.gov/dnaworks/), and manually edited by OligoAnalyzer (https://eu.idtdna.com/calc/analyzer/) correcting for GC-content, primer dimers and loop formation probabilities.

PCR products were purified by Gel Extraction Centrifugal Filter Units (Merck Millipore, Darmstadt, Germany), and dA-tailing was performed (Taq DNA polymerase, Evrogen, Russia). Constructs were ligated into a pGEM®-T Easy vector (Promega, Madison, USA). Five positive clones were selected and sequenced by Applied Biosystems 3500 Series Genetic Analyzers (ThermoFisher Scientific, Waltham, USA). The genes were double digested by restriction enzymes and ligated into the specific expression vectors listed in above table (Novagen, USA). Vectors were transformed into *Escherichia coli* BL21 (DE3) cells (ThermoFisher Scientific, Madison, USA) and ampicillin or kanamycin selected colony cultures were induced with isopropyl β-d-thiogalactopyranoside at OD 0.8-1.2 and incubated at 30°C. Cultured cells were spun down and resuspended in 50 мM Tris-HCl containing 1 mg/ml lysozyme (Sigma-Aldrich, Burlington, USA), after which 50 мM Tris-HCl containing 1 M KCl, 1% Triton X-100, 0.4% Tween20 and 2 мM PMSF was added. Cell suspensions were sonicated, centrifuged, and run over Ni-Sepharose columns (Cytiva, Marlborough, USA) using manufacturer instructions and eluted using 300 µM imidazole. Flow through fractions were checked by SDS-page. Proteins for pooled fractions were subsequently purified using DEAE Sepharose Fast Flow (Vsp1, Vlp15/16, Vlp18, Vlp5, and p41

from *B. bavariensis*), or Sephadex G-25 (p41 from *B. afzelii*) (GE Healthcare, Chicago, USA). Protein concentrations were determined by Bradford protein assay (Bradford *et al.* 1976) and set at concentration (Supplemental appendix 2).

Supplemental appendix 2. Properties of recombinant proteins solutions

Protein	*Borrelia* spp., strain	Buffer	Concentration (mg/ml)
GlpQ	*B. miyamotoi*,<br>LB2001	25 мМ Tris/HCl, pH 7.5, 0.25 M NaCl, 0.15 M imidazole, 0.1% Tween20, 50% glycerol	4.3
Vsp-1	*B. miyamotoi*,<br>LB2001	25 мМ Tris/HCl, pH 8.0, 0.05 M KCl, 50% glycerol	1.5
Vlp-5	*B. miyamotoi*,<br>LB2001	25 мМ Tris/HCl, pH 8.0, 0.05 M KCl, 50% glycerol	2.0
Vlp-15/16	*B. miyamotoi*,<br>LB2001	25 мМ Tris/HCl, pH 8.0, 0.05 M KCl, 50% glycerol	1.8
Vlp-18	*B. miyamotoi*,<br>LB2001	25 мМ Tris/HCl, pH 8.0, 0.05 M KCl, 50% glycerol	4.5
p41	*B. afzelii*,<br>pKo	PBS, pH 7.5	1.8
p41	*B. bavariensis*,<br>BPi	50 мМ Tris/HCl, pH 8.0, 0.4 M KCl, 2 M urea	0.64

Properties of the recombinant protein solutions prepared for the coating of diagnostic glass slides. Characteristics include the names of the genes, the strains and isolates they were obtained from, the buffer they are diluted in at a predetermined concentration in mg/ml.

Abbreviations: B.: *Borrelia*, GlpQ: glycerophosphodiester phosphodiesterase, KCl: Potassium chloride, M: molar, mg: milligram, ml: millilitre, mM: millimolar, NaCl: sodium chloride, p41: flagellin, PBS: Phosphate buffered saline, pH: potential of hydrogen, spp.: species, Tris/HCL: Tris(hydroxymethyl)aminomethane hydrochloride, Vlp: variable large protein, Vsp: variable small protein.

Supplemental appendix 3: Protocol of protein array preparation, procedure and read-out

Protein array preparation
To measure the IgM and IgG antibody response directed against recombinant proteins, antigens and controls were spotted in triplicates using sciFLEXARRAYER SX (Scienion AG, Berlin, Germany) on aldehyde-activated glass slides VALS (Cel Associates, Pearland, USA). The surface of the slide was divided into 12 separate test fields, each comprising ten by ten printed spots of recombinant proteins. As a printing negative control the buffer phosphate-buffered saline (PBS) was used. Purified IgM and IgG (Jackson ImmunoResearch, West Grove, USA) was used as a printing positive control series at 5μg/ml, 10μg/ml, and 50μg/ml. Cystine-3 and Cystine-5 N-hydroxysuccinimide-ester labeled bovine serum albumin (BSA) (Sigma-Aldrich, Burlington, USA) was used for boarder markers and calibration. After overnight incubation at 4°C, the slides were blocked in 0.5% BSA in PBS for 1 hour at 37°C and stored at -20°C for future use.

Protein array procedure
Slides were thawed and washed with a wash buffer (PBS with 0.05% Tween20 at pH 7.5) for 2 minutes at 37°C shaking 500rpm. Fast 12-well Frames and Fast Slide Holders (Sigma-Aldrich, Burlington, USA) were used for well-formation to create test fields on the glass slide. Wells were incubated with serum samples 1:10 in binding buffer (PBS with 5.6% BSA, 0.05% Triton X-100, 0.05% Tween20, and 0.005% NaH3 at pH 7.5) for 30 minutes at 37°C shaking 500rpm. Human sera was pooled to generated negative and positive controls, tested for the presence of antibodies against *B. miyamotoi* (experimental rGlpQ and rVmp ELISA and Western blot [1]) and *B. burgdorferi* s.l. (Liaison, Atlanta, USA). Next, wells were aspirated, washed, and incubated with a secondary antibody mixture of Cy3-conjugated goat anti-human IgM antibodies (1:20.000, 50 ng/ml) and Cy5-conjugated goat anti-human IgG antibodies (1:10.000, 100 ng/ml) (Jackson ImmunoResearch, West Grove, USA) in binding buffer for 30 minutes at 37°C shaking 500rpm. Wells were aspirated and washed. Frames were removed, slides were washed in milli-Q water, and air dried at room temperature. Finally, fluorescence read out was performed by array laser MArS (DitaBis, Pforzheim, Germany).

Protein array read-out
Images were quantified using SpotScout (DitaBis, Pforzheim, Germany) in accordance with the user manual. An Excel (Version 2016, Microsoft Corporation, USA) template was developed to process raw numeric data. To account for intra-test variation, medians of triplicates were calculated. Next, to eliminate background signal, the median negative control was subtracted from all tested samples per slide. Finally, human IgM and IgG dose-response calibration curves were made by an exponent regression model, to interpolate the antigen specific IgM- and IgG-concentrations (μg/mL) in serum [2-4].

Supplemental appendix 4: Antibody levels in BMD patients and control groups

IgM									
			Mean antibody concentration, µg/ml (quartiles)						
**Group**		**Samples, N**	**GlpQ**	**Vsp-1**	**Vlp-5**	**Vlp-15/16**	**Vlp-18**	**Any Vmp**	**Flagellin**
**BMD patients**	**Acute**	72	0.6**** (0.2-1.9)	0* (0-0.7)	0.4^{ns} (0-1.5)	0.6** (0-1.9)	0^{ns} (0-0.8)	1.8^{ns} (0.7-4.3)	1.3^{ns} (0.4-2.5)
	**Early**	66	9.7**** (3.2-19.2)	1.1**** (0.1-6.1)	3.1**** (1.1-8.4)	5.4**** (1.7-11.8)	0.7**** (0-2.4)	10.3**** (3.2-18.8)	6.3**** (2.9-12.9)
	**Late**	46	6.8**** (2.7-13.9)	1.3*** (0-6.4)	2.2**** (1.1-6.2)	5.2**** (2.1-13.1)	0.8**** (0.1-3.6)	10.0**** (4.8-16.1)	5.9**** (2.9-13)
	**Reconvalescent**	57	1.4**** (0.7-2.8)	1.3*** (0.1-3.6)	0.8*** (0.2-2)	2.2**** (0.6-3.7)	0.4**** (0-3.2)	3.8**** (2.2-6.6)	3.4**** (1.7-8)
**Cross-reactive controls**	**LB**	132	0 (0-0)	0.1 (0-0.9)	0 (0-1.4)	0.3 (0-2.2)	0 (0-0.4)	1.4 (0.2-3.7)	2.8 (0.8-6.1)
	**ID**	144	0 (0-0.9)	0.2 (0-1)	0 (0-0.7)	0.1 (0-0.7)	0 (0-0.5)	0.6 (0.1-2.2)	0.5 (0-2.3)
**Healthy blood donors**	**Endemic**	697	0.3 (0-1.2)	0.6 (0-2.7)	0.4 (0-1.8)	0 (0-1.5)	0.1 (0-1.2)	2.1 (0.5-4.3)	1.3 (0.3-2.9)
	**Non-endemic**	183	0.1 (0-0.9)	0 (0-0.2)	0 (0-0.6)	0.3 (0-1.1)	0 (0-0.4)	0.9 (0.2-2.2)	0.3 (0-1.2)
**Any control**		1156	0.1 (0-1)	0.2 (0-1.8)	0.2 (0-1.4)	0.1 (0-1.4)	0 (0-0.9)	1.5 (0.3-3.6)	1.1 (0.1-2.9)

IgG									
			Mean antibody concentration, µg/ml (quartiles)						
**Group**		**Samples, N**	**GlpQ**	**Vsp-1**	**Vlp-5**	**Vlp-15/16**	**Vlp-18**	**Any Vmp**	**Flagellin**
**BMD patients**	**Acute**	72	0.1*** (0-0.5)	0^{ns} (0-0.4)	0.2**** (0-0.3)	0^{ns} (0-0.6)	0^{ns} (0-0.1)	0.4^{ns} (0.1-2.5)	0.4^{ns} (0.1-1.7)
	**Early**	66	0.5****	0.1^{ns}	0.2****	0.27****	0^{ns}	2.7****	1.9****

			(0-7.9)	(0-2.1)	(0.1-2.6)	(0-6)	(0-0.3)	(0.3-16.7)	(0.4-8.7)
	**Late**	46	15.4****	0.6**	1.4****	14.76****	0*	22.4****	1.9****
			(3.3-29.1)	(0-4.2)	(0.2-6.4)	(1.5-37.2)	(0-1.3)	(13.9-42.8)	(1.2-12.4)
	**Reconvalescent**	57	11.0****	0.5*	0.6****	6.8****	0*	16.2****	1.2**
			(1.7-26.5)	(0-13.3)	(0-4.3)	(0.1-21.5)	(0-0.8)	(6.2-31.5)	(0.1-5.1)
**Cross-reactive controls**	**LB**	132	0.1	0.1	0.4	0.675	0.1	1.4	3.6
			(0-0.9)	(0-0.8)	(0-4.8)	(0-8.7)	(0-1.3)	(0.4-13.7)	(1.2-15.9)
	**ID**	144	0.1	0	0	0	0	0.1	0.4
			(0-0.9)	(0-0.3)	(0-0)	(0-0.1)	(0-0)	(0-0.9)	(0-2.4)
**Healthy blood donors**	**Endemic**	697	0	0.7	0	0	0	1.4	0.5
			(0-0.1)	(0-2.4)	(0-0)	(0-0.7)	(0-0.1)	(0.4-3.3)	(0-1.7)
	**Non-endemic**	183	0	0	0	0	0	0.2	0.1
			(0-0)	(0-0)	(0-0)	(0-0.1)	(0-0.2)	(0-0.8)	(0-1.2)
**Any control**		1156	0	0.2	0	0	0	0.9	0.5
			(0-0.2)	(0-1.6)	(0-0)	(0-0.6)	(0-0.2)	(0.1-2.9)	(0-2.3)

Median values and quartiles of IgM and IgG concentration in *B. miyamotoi* disease (BMD) sample groups: BMD acute (0-4 days after disease onset), BMD early (5-25 days after disease onset), BMD late (26-149 days after disease onset), and reconvalescent (150-499 days after disease onset), compared to disease controls (culture proven Lyme borreliosis (LB) patients), cross-reactive controls with other infectious diseases (ID, patients with serological proven leptospirosis, syphilis, CMV, EBV or HSV I/II infection and culture proven *H. influenzae* bacteraemia), *Ixodes* tick endemic healthy blood donors (from Russia and the Netherlands), and *Ixodes* tick non-endemic healthy controls (from Russia and Norway).

Significance was calculated with Mann-Whitney tests by comparing BMD sample groups to combined controls (combined samples from all control groups). Ns, non-significant; * $p < 0.05$; ** $p < 0.01$; *** $p < 0.001$; **** $p < 0.0001$.

Abbreviations: BMD: *Borrelia miyamotoi* disease, GlpQ: glycerophosphodiester phosphodiesterase, Ig: immunoglobulin, ID: infectious diseases, LB: Lyme borreliosis, N: total amount of samples, Vlp: variable large protein, Vmp: variable major protein, Vsp: variable small protein.

Supplemental appendix 5: ROC curves for IgM and IgG against individual B. miyamotoi antigens in BMD patients and control groups

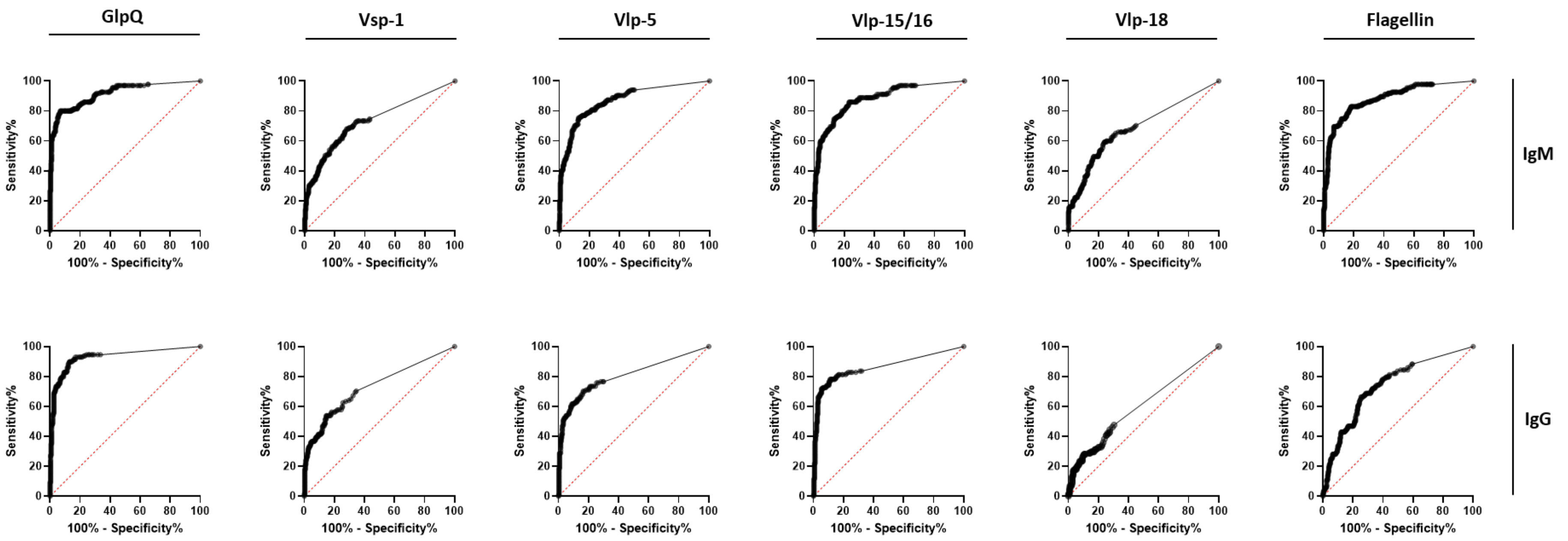

Receiver operating characteristic curves for the binary classification of IgM and IgG antibody concentration against the recombinant *B. miyamotoi* antigens in BMD patients and control groups. The sensitivity is determined in the BMD patients and the specificity in 280 healthy controls, comprising Russian endemic and Russian and Norwegian non-endemic healthy controls. Cut-offs for both IgM and IgG were set at 5 µg/ml with a specificity > 95% for IgM and a specificity > 93% for IgG.

Abbreviations: B.: *Borrelia*, BMD: *Borrelia miyamotoi* disease, GlpQ: glycerophosphodiester phosphodiesterase, Ig: immunoglobulin, ROC: Receiver operating characteristic, Vlp: variable large protein, Vsp: variable small protein.

Supplemental appendix 6: Diagnostic power of the measurements of specific IgM- and IgG-antibodies represented as Area Under the Curve of Receiver Operating Characteristics

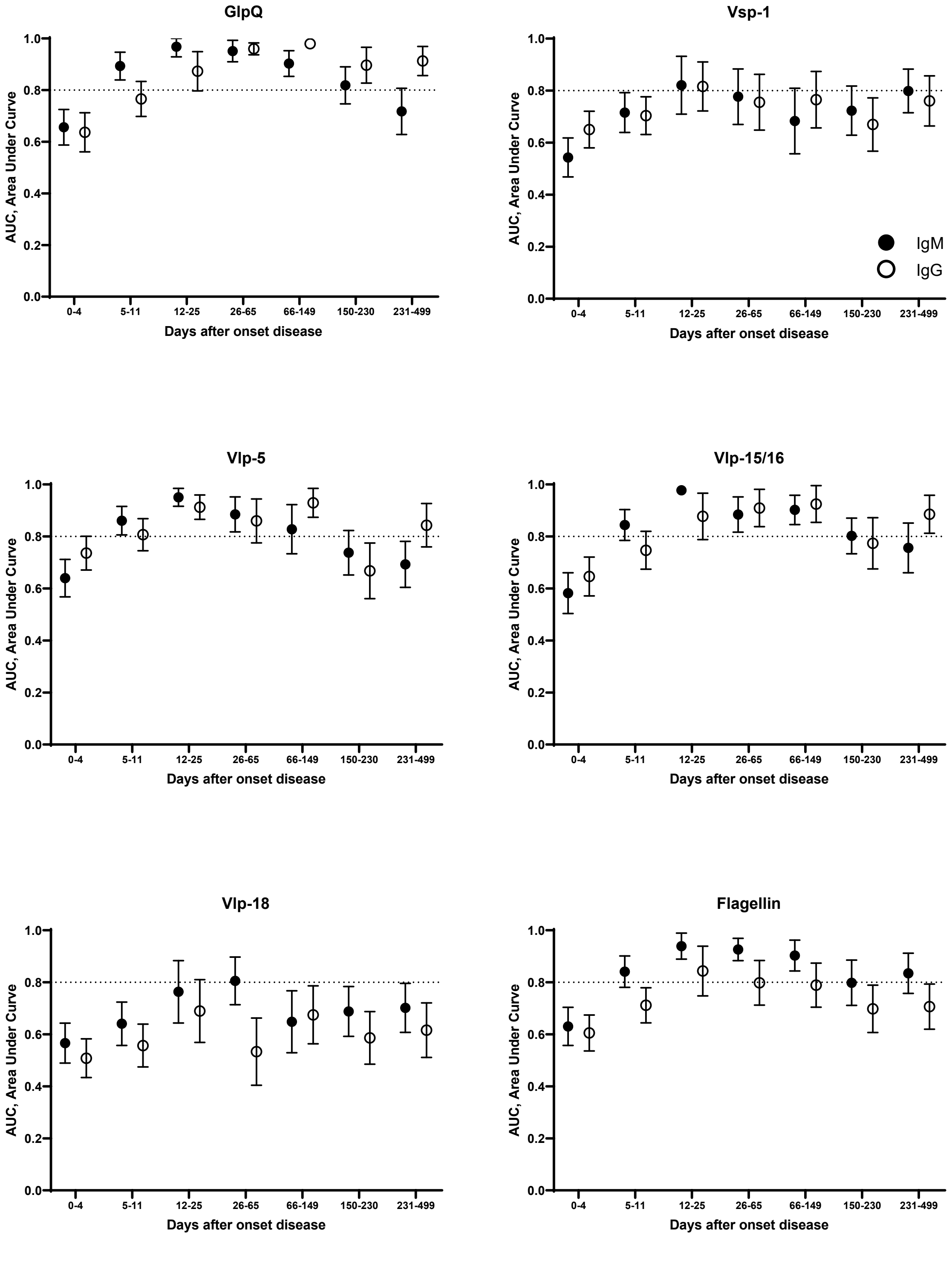

The diagnostic power of the measurements of specific IgM- and IgG-antibodies against individual *B. miyamotoi* antigens represented as Area Under the Curve with standard error of the mean of Receiver Operating Characteristics in BMD patient over time.

Abbreviations: AUC: area under the curve, B.: *Borrelia*, BMD: *Borrelia miyamotoi* disease, GlpQ: glycerophosphodiester phosphodiesterase, Ig: immunoglobulin, ROC: Receiver operating characteristic, Vlp: variable large protein, Vsp: variable small protein.

Supplemental appendix 7: Sensitivity and specificity of individual and combination of antigens of the protein array in BMD patients over time

**IgM**										
	**Individual antigens Sensitivity (95% CI)**							**Combination of antigens Sensitivity (95% CI)**		
	**GlpQ**	**Vsp-1**	**Vlp-5**	**Vlp-15/16**	**Vlp-18**	**p41**	**Any Vmp**	**GlpQ or any Vmp**	**GlpQ and any Vmp**	**GlpQ and (any Vmp or p41)**
**Specificity**	98.2%	97.9%	98.9%	97.1%	98.9%	96.4%	94.3%	93.2%	99.3%	99.3%
**(95% CI)**	(95.9-99.4)	(95.4-99.2)	(96.9-99.8)	(94.5-98.8)	(96.9-99.8)	(93.5-98.3)	(90.9-96.7)	(89.6-95.9)	(97.4-99.9)	(97.4-99.9)
**BMD time-window, days**										
0-4	12.5	4.2	11.1	11.1	8.3	11.1	22.2	31.9	2.8	2.8
	(6.6-22.4)	(1.3-12.2)	(5.6-20.7)	(5.6-20.7)	(3.8-17.4)	(5.6-20.7)	(14.1-33.3)	(22.2-43.6)	(0.7-10.5)	(0.7-10.5)
5-11	36.5	20.6	38.1	46.0	14.3	49.2	63.5	76.2	50.8	61.9
	(51.0-74.4)	(12.3-32.4)	(27.0-50.6)	(34.1-58.4)	(7.6-25.3)	(37.1-61.4)	(51.0-74.4)	(64.1-85.1)	(38.6-62.9)	(49.4-73.0)
12-25	70.0	35.0	35.0	80.0	20.0	75.0	85.0	95.0	60.0	70.0
	(47.2-85.9)	(17.6-57.5)	(17.6-57.5)	(57.1-92.3)	(7.7-42.9)	(52.1-89.2)	(62.3-95.1)	(71.6-99.3)	(37.9-78.6)	(47.2-85.9)
26-65	65.4	26.9	23.1	42.3	23.1	57.7	69.2	84.6	50.0	65.4
	(45.6-81.0)	(13.4-46.8)	(10.7-42.9)	(25.1-61.6)	(10.7-42.9)	(38.4-74.9)	(49.4-83.9)	(65.4-94.1)	(31.6-68.4)	(45.6-81.0)
66-149	53.8	26.9	26.9	42.3	7.7	53.8	57.7	76.9	34.6	50.0
	(35.0-71.7)	(13.4-46.8)	(13.4-46.8)	(25.1-61.6)	(1.9-26.2)	(35.0-71.7)	(38.4-74.9)	(57.1-89.3)	(19.0-54.4)	(31.6-68.4)
150-230	19.5	17.1	4.9	14.6	12.2	31.7	36.6	46.3	9.8	12.2
	(10.0-34.5)	(8.3-31.8)	(1.2-17.6)	(6.7-29.0)	(5.1-26.2)	(19.3-47.3)	(23.4-52.2)	(31.8-61.5)	(3.7-23.4)	(5.1-26.2)
231-499	11.4	8.6	0	14.3	11.4	40.0	22.9	31.4	2.9	5.7
	(4.3-26.9)	(2.8-23.5)	(na)	(6.1-30.1)	(4.3-26.9)	(25.3-56.8)	(11.8-39.6)	(18.3-48.4)	(4.0-17.8)	(1.4-20.3)

**IgG**			
	**Individual antigens Sensitivity (95% CI)**		**Combination of antigens Sensitivity (95% CI)**

	GlpQ	Vsp-1	Vlp-5	Vlp-15/16	Vlp-18	p41	Any Vmp	GlpQ or any Vmp	GlpQ and any Vmp	GlpQ and (any Vmp or p41)
**Specificity (95% CI)**	97.5% (94.9-99.0)	98.6% (96.4-99.6)	99.3% (97.4-99.9)	96.8% (94.0-98.5)	98.2% (95.9-99.4)	93.2% (89.6-95.9)	94.6% (91.3-97.0)	92.9% (89.2-95.6)	99.3% (97.4-99.9)	99.3% (97.4-99.9)
**BMD time-window, days**										
0-4	11.1 (5.6-20.7)	2.8 (0.7-10.5)	2.8 (0.7-10.5)	9.7 (4.7-19.1)	4.2 (1.3-12.2)	9.7 (4.7-19.1)	13.9 (7.6-24.0)	19.4 (11.8-30.3)	5.6 (2.1-13.9)	6.9 (2.9-15.7)
5-11	22.2 (13.6-34.2)	12.7 (6.5-23.5)	12.7 (6.4-23.5)	20.6 (12.3-32.4)	9.5 (4.3-19.7)	27.0 (17.4-39.3)	33.3 (22.8-45.8)	41.3 (29.8-53.8)	14.3 (7.6-25.3)	14.3 (7.6-25.3)
12-25	40.0 (21.4-62.1)	15.0 (0.5-37.7)	15.0 (4.9-37.7)	40.0 (21.4-62.1)	5.0 (0.7-28.4)	50.0 (29.3-70.7)	55.0 (33.5-64.6)	75.0 (52.1-89.2)	20.0 (7.7-42.9)	30.0 (14.1-52.8)
26-65	61.5 (42.0-78.0)	23.1 (10.7-42.9)	30.8 (16.1-50.6)	65.4 (45.6-81.0)	15.4 (5.9-34.6)	34.6 (19.0-54.4)	84.6 (65.4-94.1)	88.5 (69.6-96.2)	57.7 (38.4-74.9)	61.5 (42.0-78.0)
66-149	80.8 (61.2-91.8)	19.2 (8.2-38.8)	26.9 (13.4-46.8)	73.1 (53.2-86.6)	3.8 (0.5-22.9)	30.8 (16.1-50.6)	88.5 (69.6-96.2)	92.3 (73.8-98.1)	76.9 (57.1-89.3)	80.8 (61.2-91.8)
150-230	73.2 (57.7-84.5)	26.8 (15.5-42.3)	22.0 (11.8-37.1)	53.7 (28.5-68.2)	7.3 (2.4-20.4)	24.4 (13.6-39.8)	73.2 (57.7-84.5)	87.8 (73.8-94.9)	58.5 (43.1-72.5)	61.0 (45.4-74.6)
231-499	60.0 (43.2-74.7)	25.7 (13.9-42.6)	25.7 (13.9-42.6)	62.9 (45.9-77.1)	2.9 (0.4-17.8)	25.7 (13.9-42.6)	80.0 (63.5-90.2)	91.4 (76.5-97.2)	48.6 (32.7-64.8)	51.4 (35.2-67.3)

Sensitivity and specificity with 95%CI for IgM and IgG given for individual antigens and combination of antigens. The sensitivity is shown in BMD patient for each time window in days. The specificity was determined in 280 healthy controls, comprising Russian endemic and Russian and Norwegian non-endemic healthy controls. Cut-offs for both IgM and IgG were set at 5 µg/ml with a specificity > 95% for IgM and a specificity > 93% for IgG. Combinations of antigens are shown as either ‘or’ or ‘and’. ‘Or’ indicates either antigen-specific antibody levels being increased. ‘And’ indicates both antigen-specific antibody levels being increased.

Abbreviations: BMD: *Borrelia miyamotoi* disease, CI: confidence interval, GlpQ: glycerophosphodiester phosphodiesterase, Ig: immunoglobulin, p41: flagellin, Vlp: variable large protein, Vmp: variable major protein, Vsp: variable small protein.

References

1. Koetsveld J, Kolyasnikova NM, Wagemakers A, et al. Serodiagnosis of Borrelia miyamotoi disease by measuring antibodies against GlpQ and variable major proteins. Clin Microbiol Infect **2018**; 24(12): 1338 e1- e7.
2. Ardizzoni A, Capuccini B, Baschieri MC, et al. A protein microarray immunoassay for the serological evaluation of the antibody response in vertically transmitted infections. Eur J Clin Microbiol Infect Dis **2009**; 28(9): 1067-75.
3. Rubina AY, Dementieva EI, Stomakhin AA, et al. Hydrogel-based protein microchips: manufacturing, properties, and applications. Biotechniques **2003**; 34(5): 1008-14, 16-20, 22.
4. Daly DS, White AM, Varnum SM, Anderson KK, Zangar RC. Evaluating concentration estimation errors in ELISA microarray experiments. BMC Bioinformatics **2005**; 6: 17.
